# Analysis of Rheumatic Heart Disease Mortality in the Chinese Population: A JoinPoint and Age–Period–Cohort Study

**DOI:** 10.3390/ijerph19169872

**Published:** 2022-08-10

**Authors:** Jiameng Cui, Xinru Guo, Xin Yuan, Hao Wu, Ge Yu, Biao Li, Changgui Kou

**Affiliations:** 1 Department of Epidemiology and Biostatistics, School of Public Health, Jilin University, No. 1163 Xinmin Street, Changchun 130021, China; 2 Department of Social Medicine and Health Management, School of Public Health, Jilin University, No. 1163 Xinmin Street, Changchun 130021, China

**Keywords:** rheumatic heart disease, mortality, JoinPoint regression model, age–period–cohort model, China

## Abstract

(1) Background: We aimed to analyze rheumatic heart disease (RHD) mortality trends in China’s urban and rural areas and to determine the roles of age, period, and cohort effects. (2) Methods: Based on mortality data extracted from the China Health Statistics Yearbook, we calculated the crude mortality rate of RHD. Age–adjusted rates were computed by the direct method using the 2020 census as the standard population. The annual percentage change (APC) and average annual percentage change (AAPC) were determined by the JoinPoint regression model. The age–period–cohort model was used to estimate the effects of age, period, and cohort. (3) Results: From 2006 to 2020, the general trend in RHD standardized mortality declined. The RHD mortality rate was higher in rural than in urban areas and among females than males. The elderly (over 60 years old) were at high risk for RHD deaths in China. The age effect increased with age, and the cohort effect showed a declining trend as chronology grew, but the period effect was not significant. (4) Conclusions: China has achieved great success in RHD, but RHD mortality may increase with age. Compared with the period effect, age and cohort effects dominated the risk of RHD deaths.

## 1. Introduction

Rheumatic heart disease (RHD) is one of the sequelae of acute rheumatic fever [1]. Upon progression, RHD can result in cardiac calcification and even heart failure, and can have a severe negative impact on health and quality of life [2]. Global RHD morbidity and mortality declined in the 21st century, along with improvements in housing congestion and environmental pollution [3]. RHD morbidity and mortality rates in developed countries are almost 0, and RHD is currently concentrated in low–income countries and poverty groups of high–income countries [4,5]. In 2015, the three countries with the most deaths due to RHD were estimated to be India, China, and Pakistan [6]. By 2020, the Chinese RHD death rate was still high, at 3.18/100,000 in urban areas and 3.81/100,000 in rural areas, far above the rates of common malignant tumors, such as nasopharyngeal carcinoma [7]. An aging society and the shifting medical environment are affecting the prevalence of RHD in China. However, there is little available literature providing evaluation and guidance on public health interventions for RHD in China. Sun’s research focused solely on overall trends in cardiovascular disease mortality and years of life lost [8], and the trends in RHD mortality in China were vague. Therefore, it is critical to understand the integrated role of causative agents and sanitary measures and to propose targeted policies.

Using data from only one cross–section tends to confound the relationship between age, period, and cohort. For example, when interpreting the effect of age on RHD, period and birth cohort differences must be considered. However, the JoinPoint Regression Model can independently assess the pattern of changes in RHD mortality, and the Age–Period–Cohort model (APC model) can decompose the effects of age, period, and birth cohort factors on the risk of RHD death.

The goal of this study was to provide a reference base for investigating the RHD mortality trend and performing precise sanitary work. We applied the JoinPoint Regression Model to explore trends in RHD mortality by sex and area from 2006 to 2020. We also used the APC model to quantify the effect coefficients.

## 2. Methods

### 2.1. Data Sources

Between 2006 and 2020, this study collected age, sex, and regional (urban, rural) RHD mortality data from the China Health Statistics Yearbook under the cause of death surveillance system. The system is a continuous, nationally representative mortality surveillance program elucidating the residents’ distribution of deaths and is administered by the National Health Commission (previously the National Health and Family Planning Commission) Center of Health Information and Statistics (CHIS). The integrated national mortality surveillance system provided a comprehensive death registration and cause of mortality monitoring system for the whole country, which increased the surveillance population to 24% of the Chinese population. Furthermore, the number of surveillance points, each of which covered a district or county, increased from 161 to 605. Hence, the data represent China well. We adopted the 10th revision of the International Classification of Diseases to code for chronic rheumatic heart disease [ICD10:I05–I09].

### 2.2. Data Analysis

The crude mortality rate for RHD was calculated separately for urban and rural areas, as well as for males and females. Using the seventh census data in 2020 as the standard population, age–adjusted mortality rates in all subgroups were calculated. Since the mortality rate of RHD under 30 years old was virtually 0, and the cause of death was more complicated in patients aged 80 years and above, we considered the population aged 30–79 years in China as participants.

#### 2.2.1. JoinPoint Regression Model

To determine the magnitude of the secular trends for the RHD mortality rate, the JoinPoint Regression Program 4.9.0.0 (available through the National Cancer Institute’s Surveillance Research Program) was applied to fit the trend of RHD mortality and compute the annual percentage change (APC) and average annual percentage change (AAPC). The model truncated and divided age into five–year intervals (30–34, 35–39,…, 75–79), with up to three turning points based on the incorporated data. In this model, the mortality rates were also log–transformed, and the 95% confidence intervals (95% CIs) were set to 0.05 by the Monte Carlo permutation method.

#### 2.2.2. Age–Period–Cohort Model

Based on a Poisson distribution, the age–period–cohort model (APC model) is frequently employed in studies of chronic noncommunicable disease morbidity and mortality [9]. Because there is a linear relationship between age, period, and cohort (cohort=period−age), the APC model cannot identify the unique estimates of the three effect parameters. To address this issue, Fu proposed the intrinsic estimator (IE) method over the estimation function method [10]; subsequently, IE was later demonstrated by Yang et al. to produce convergent and exclusive estimates [11]. Therefore, in this study, the APC model combined with IE was performed to roughly calculate the risk parameter of RHD death. We appropriately divided the data into ten age groups and three period groups, with five–year group intervals. The birth cohort was grouped into twelve cohort groups based on the linear relationship between birth cohort, period, and age. We also used the years 2008, 2013, and 2018 to substitute for the three period groups in the APC model to avoid data overlap between adjacent cohorts. Finally, our study implemented the APC model with Stata 14.0 (StataCorp, College Station, TX, USA).

## 3. Results

**Figure 1:**
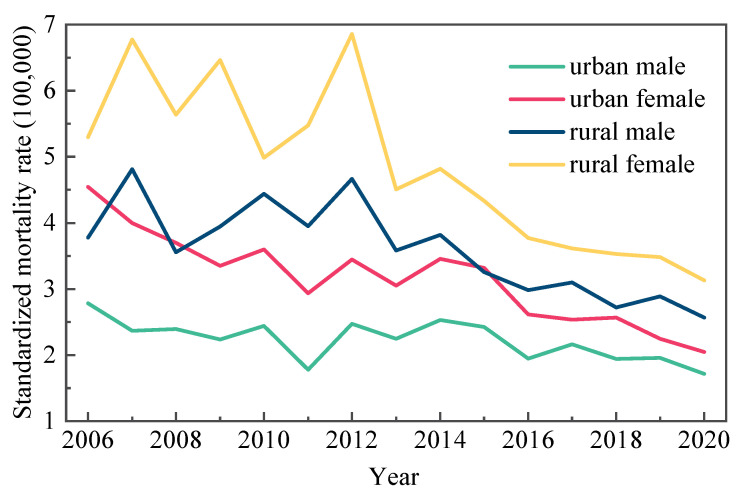
Changes in the standardized mortality rate of RHD in urban and rural areas by sex in China (1/100,000).

During the period 2006–2020, the RHD death rate in urban men/women and rural men/women declined. Rural regions had a higher average standardized mortality rate (4.2/100,000) than urban areas (2.7/100,000). As shown in Figure 1, males and females in urban areas showed consistently lower age–adjusted death rates than males and females in rural areas.

From 2006 to 2020, the JoinPoint analysis found a significant decrease in RHD standardized and crude mortality rates. When comparing the JoinPoint regression results for standardized and gross mortality rates, the age–adjusted and crude mortality rates for urban men and women indicated no difference. Between 2006 and 2012, the age–adjusted mortality rate for rural males and females did not present a statistically significant change. After 2012, APC fell by 6.0% and 7.3%, respectively. While the crude mortality rate continued to decline by 2.0% and 3.2% per year from 2006 to 2020 (Table 1 and Table 2).

Using the JoinPoint regression results by age category, the age–specific mortality rates of the four subgroups showed a marked decreasing trend year by year, and we found that most of the turning points for the urban residents occurred in 2015 and 2017, for the rural mostly in 2012. However, the decline was significantly smaller in the elderly (60 years and older), and it even grew at an annual rate of 0.2% and 2.0% in the age groups of 70–74 and 75–79 urban males, respectively (p>0.05). In particular, RHD mortality fell annually (p<0.05) in most age groups of urban men (30–69 years) and all age groups of urban women, and most middle–aged rural women (35–39, 40–44, 45–49, and 55–59 years) showed a decrease of 10% or more in the annual percentage change (Table 3 and Table 4).

**Figure 2:**
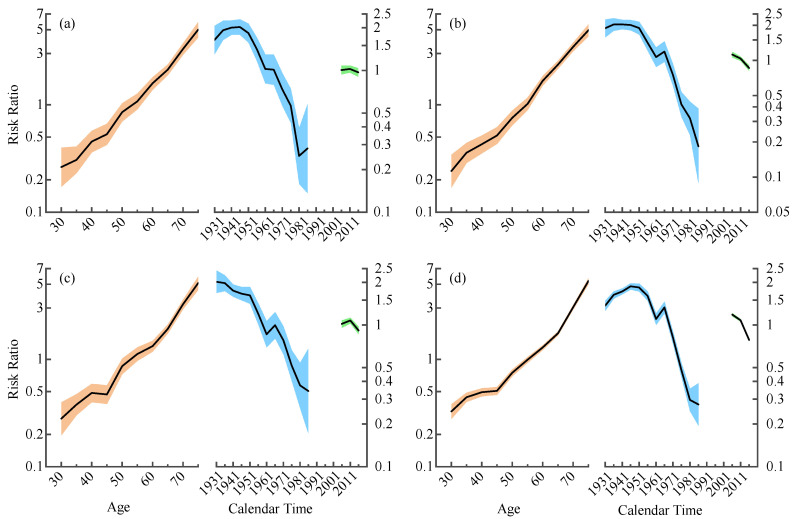
Multivariable age–period–cohort analysis of RHD mortality rates by region and sex in China from 2006 to 2020. For the 3 line graphs, the left lines represent age effect, the middle lines are the estimated birth cohort effect, and the short lines refer to the period effects (risk ratio). The shaded areas surrounding the lines represent the 95% confidence intervals. (**a**) Urban male, (**b**) urban female, (**c**) rural male, (**d**) rural female.

**Figure 3:**
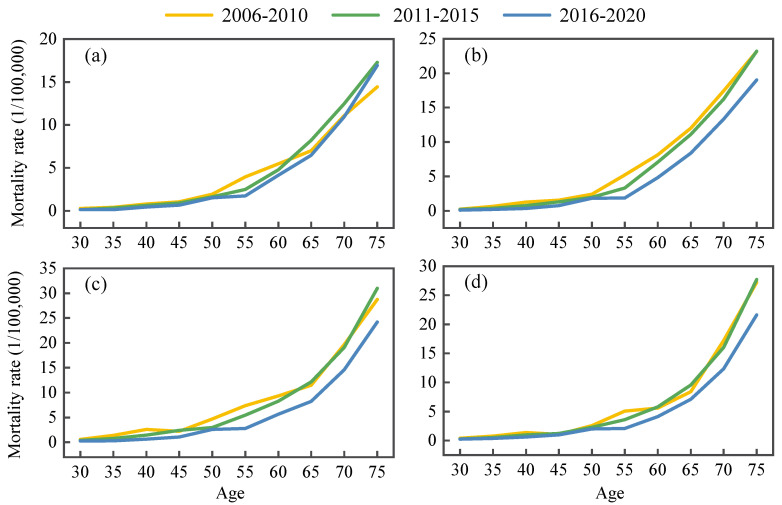
Mortality rates of RHD in China from 2006 to 2020. Mortality data were collected from the Disease Surveillance Points system of China. Age analyses were from 30 to 79, with every 5–year interval as one age. (**a**) Urban male, (**b**) urban female, (**c**) rural male, (**d**) rural female.

**Figure 4:**
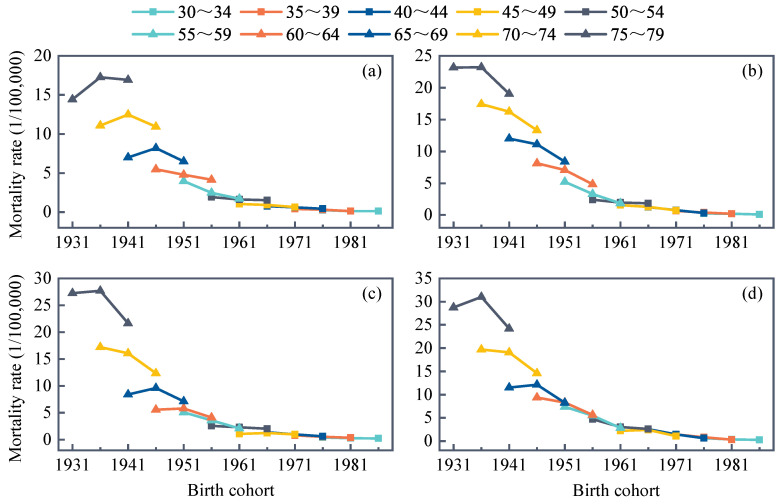
Mortality rates of RHD in China among those aged 30 to 79 years. Mortality data were collected from the Disease Surveillance Points system of China. Birth cohorts analyses were from 1931 to 1990, with every 5–year interval as one cohort. (**a**) Urban male, (**b**) urban female, (**c**) rural male, (**d**) rural female.

The APC model illustrated the results of the age, period, and cohort effects in urban and rural residents for both genders separately and demonstrated that age and birth cohort were the main risks for RHD death (Figure 2). Figure 2 showed that age was the strongest factor affecting RHD mortality among the three factors studied, and the older patients with RHD had a greater risk of death. Moreover, statistically significant cohort effects arose while there were no period effects. The age effect increased with age, and period–specific mortality was plotted with age to assess the age effect more visually (Figure 3). This result indicated that, during the three periods of 2006–2010, 2011–2015, and 2016–2020, age-specific mortality rates for urban and rural populations of different sexes and age subgroups were relatively low and stable up to age 50. Then, they consistently grew in the 50–75 age range and peaked at 75–79 years old. After controlling for age and cohort effects, the period effect coefficient fluctuated inconspicuously and fell overall. Variations in cohort effect were more complex, showing a slow change followed by a decline before the 1963 birth cohort. The 1964–1968 birth cohort groups generally illustrated a slight upward trend, with a more distinct downward trend after 1969. In summary, the whole coefficient of the cohort effect dropped from 1931 to 1990. Undeniably, in the same birth cohort, mortality rates for urban males and rural males/females showed an upward and then a downward trend at ages 65–69 and 75–79 years, respectively (Figure 4). Young adults had a lower risk of death from RHD than older people.

## 4. Discussion

To the best of our knowledge, this is the first study to exclusively utilize JoinPoint regression model to analyze trends in RHD mortality in China and to decompose the effects of age, period, and cohort by the APC model for urban and rural areas and men and women. The findings indicated a downward trend in the RHD standardized mortality rate from 2006 to 2020, with obvious regional differences. In the same years, the rural standardized rate was higher than the urban rate, and the female rate was higher than the male rate; however, the discrepancies between urban and rural areas gradually shrank. In the early 20th century, the timely use of antibiotics, such as penicillin, helped avoid subsequent rheumatic fever and rheumatic heart disease. It also dramatically reduced the prevalence and severity of RHD in China and improved the patients’ quality of life, with approximately 70% of acute rheumatic fever patients recovering within 2–3 months [12]. Sex differences in RHD mortality may be associated with female autoimmune susceptibility and the intensity of streptococcal exposure [13,14]. In addition, improving health awareness and education has kept people away from risk factors. From 1993 to 1995, the Chinese government conducted regular surveys on the prevalence of RHD, as well as improved health education for children and adolescents with streptococcal pharyngitis and rheumatic fever [15]. Meanwhile, urban and rural schooling levels improved dramatically from 2006 to 2020, particularly in rural schooling [16]. All of these factors contributed to a better quality of health services for Chinese residents regarding RHD. To some extent, RHD prevention and control measures have achieved some success.

We gathered national data on RHD aged 30–79 years from China’s largest and best–accessible program. The massive sample size of the surveillance system covering 24% of China’s population, over 300 million people, made the data more representative and allowed us to describe trends in RHD in urban and rural areas by gender from 2006 to 2020. By comparing the results of the JoinPoint analysis of standardized and crude mortality, we concluded that there were no differences between urban males and urban females. The standardized mortality rates for rural males and rural females changed slowly from 2006 to 2012 and decreased by 6.0% and 7.3% per year after 2012, respectively. The crude mortality rates of rural areas showed a linear downward trend, with the standardized mortality rates declining more than the crude mortality rates. This implied that age may be an influential role in RHD mortality. Consistent with the findings of Cheng, the mortality distribution may be related to age composition [17]. Our study also found that the age–specific mortality rates decreased year by year in the four subgroups, but the percentage reduction was less for the elderly and even indicated an increasing trend for urban males over 70 years old. Many causes of death are related to age–related factors. The physical functions of the aged are diminished, and they are more susceptible to a variety of underlying disorders and significant consequences. Moreover, most RHD patients die as a direct result of chronic rheumatic heart disease combined with some more serious complications, such as respiratory infections, cardiac insufficiency, acute pulmonary edema, etc., which seriously threaten patients’ health [18]. In particular, China is facing rapid population aging. The number of people over 65 years old is expected to exceed 200 million in 2022, and China will enter a “deeply aging society” [19]. The elderly population in China is likely at an increased risk of RHD death and should be the focus of efforts to reduce RHD mortality.

The APC model discovered that age, period, and cohort effects affected all four subgroups, with age and birth cohort effects being significant. The age effect affecting RHD mortality is the most obvious among age, period, and birth cohort effects. With the exception that most older individuals are less physically fit than their younger counterparts, there are a number of other reasons to explain this finding. For instance, RHD usually does not cause acute mortality and may be insidious at onset, showing no clinical signs, so that many patients do not actively seek treatment due to poverty and limited medical knowledge, or even acceptance of therapy which always takes several years after the stage of severe valve lesions and comorbidities [20]. At the same time, the prevalence of asymptomatic RHD is distinctly higher than that of symptomatic RHD [21]. Perhaps the illness in the elderly population was already established before economic improvement or antibiotic administration. As for period effect, the three successive Medicare systems established after the 1990s appeared to explain the overall slow decline in the period effect coefficient. In addition, the decreasing trend of the mortality risk ratio in 2011–2020 is more consistent with the results of age–specific mortality JoinPoint regression, probably owing to the fact that the health system reform in China, launched in 2009, was designed to provide equal access to basic health care for all citizens and residents [22]. Chinese residents’ access to medical insurance expanded from 22.1% to 95.1% in 2013 [23], and government subsidies per capita have increased more than fivefold in 2018 compared with 2009 [24]. While the decline was not significant, it may indicate that the Chinese health department should fortify the protection of vulnerable populations. The risk varied by birth cohort, and we argue that there was an overall downward trend in the cohort effect, with the later birth cohort having a lower risk of RHD death. On the one hand, RHD is heavily influenced by socioeconomic status [25]. The population’s education rate is gradually increasing, and its socioeconomic status is generally improving, thus growing the health reserves of the younger generation and reducing the risk of RHD death [26]. On the other hand, it is associated with a rapidly developing national economy and daily improvements in living conditions. Because RHD is a disease of poverty, overcrowding, and poor sanitation, studies have demonstrated a correlation between the Gini coefficient and RHD prevalence [27,28]. Reducing exposure to pathogenic factors early in embryonic development will substantially lower the adverse effects in future life [29,30]. It has also been shown that the consumption of soybean oil and eggs can inhibit rheumatic processes [31]. Most RHD patients are malnourished with a low BMI and mid–upper arm circumference [25,32].

In short, improving living conditions, conducting public education, and receiving early antibiotic treatment may help lay a solid foundation for public health efforts in Chinese RHD. However, without sustained, intensive, and targeted public health interventions, it is difficult to guarantee that rheumatic heart disease mortality will continue to fall and that the aim of rheumatic heart disease eradication will be met. It is likely to be attributed to neglect apparently in recent decades, with multifactorial reasons including underappreciated morbidity and mortality burden, underestimated economic burden, lack of sustainable investment and so on [33]. Unlike the global disease burden of RHD, which is concentrated in females [34], the JoinPoint analysis and APC model results in our study implied that elderly populations in China are at risk of life expectancy reduction from RHD, so attempts to control RHD mortality should concentrate on these individuals. The Chinese health system is facing a progressively increasing challenge stemming from RHD mortality. Consequently, the primary healthcare system’s prevention strategies for RHD death have to be adjusted properly in order to maximize health service efficiency and minimize mortality burden. Previous study suggested that echocardiographic screening combined with health checkups should be conducted in high–risk groups, and a registry with regular follow–up should be established [35,36].

However, this study still has some limitations. First, there is no comprehensive summary of the 2006–2020 population under study that includes age, sex, type of RHD, concomitant diseases, and other features. Second, this study was subject to data quality control. Although the data source was reliable, remote areas inevitably have incomplete reporting and other circumstances [37]. The effect of morbidity on mortality in RHD in China has not been considered in the analysis of influencing factors in this study, and morbidity may have an effect on mortality trends, which also provides ideas for follow–up studies.

## 5. Conclusions

Overall, the trend of RHD mortality has decreased. To some extent, China’s RHD mortality prevention and control measures have achieved some success in the past decades. However, there remains a long path ahead. The elderly (above 60 years old) represent a risk group of RHD mortality. The period effect of RHD mortality seems to slightly decrease. Compared to earlier birth cohorts, RHD mortality risks were decreased in later birth cohorts. Aging may increase the mortality burden of RHD, emphasizing attention should be paid to predictors and factors in RHD mortality for further precise prevention and control.

## Figures and Tables

**Table 1:** AAPC and APC of standardized mortality rate of RHD among urban and rural men and women in China.

	2006–2020 Years		Period 1		Period 2		Period 3	
	AAPC (%)	95%CI	Years	APC (%)	Years	APC (%)	Years	APC (%)
Urban male	$-2.1{\;}^{*}$	(−3.4,−0.7)	2006–2020	$-2.1{\;}^{*}$				
Urban female	$-5.1{\;}^{*}$	(−9.3,−0.7)	2006–2011	$-6.6{\;}^{*}$	2011–2014	3.6	2014–2020	$-8.0{\;}^{*}$
Rural male	$-3.1{\;}^{*}$	(−5.6,−0.5)	2006–2012	1.0	2012–2020	$-6.0{\;}^{*}$		
Rural female	$-4.4{\;}^{*}$	(−6.9,−1.8)	2006–2012	−0.4	2012–2020	$-7.3{\;}^{*}$		

* Indicates statistically significant (*p* < 0.05). APC (annual percentage change); AAPC (average annual percentage change).

**Table 2:** AAPC and APC of the crude mortality rate of RHD among urban and rural men and women in China.

	2006–2020 Years		Period 1		Period 2		Period 3	
	AAPC (%)	95%CI	Years	APC (%)	Years	APC (%)	Years	APC (%)
Urban male	$-1.7{\;}^{*}$	(−3.1,−0.4)	2006–2020	$-1.7{\;}^{*}$				
Urban female	$-4.7{\;}^{*}$	(−8.0,−1.3)	2006–2011	$-7.6{\;}^{*}$	2011–2014	4.9	2014–2020	$-6.8{\;}^{*}$
Rural male	$-2.0{\;}^{*}$	(−3.4,−0.6)	2006–2020	$-2.0{\;}^{*}$				
Rural female	$-3.2{\;}^{*}$	(−4.9,−1.5)	2006–2020	$-3.2{\;}^{*}$				

* Indicates statistically significant (*p* < 0.05). APC (annual percentage change); AAPC (average annual percentage change).

**Table 3:** Temporal trend analysis of age–specific mortality rates for RHD in urban Chinese residents aged 30–79 years, 2006–2020.

Age	Period	Urban Male		Period	Urban Female	
		APC (%, 95%CI)	AAPC (%, 95%CI)		APC (%, 95%CI)	AAPC (%, 95%CI)
30–34	2006–2020	$-7.4{\;}^{*}\;\left(-12.8,-1.7\right)$	$-7.4{\;}^{*}\;\left(-12.8,-1.7\right)$	2006–2020	$-7.2{\;}^{*}\;\left(-12.4,-1.6\right)$	$-7.2{\;}^{*}\;\left(-12.4,-1.6\right)$
35–39	2006–2020	$-8.6{\;}^{*}\;\left(-13.0,-3.9\right)$	$-8.6{\;}^{*}\;\left(-13.0,-3.9\right)$	2006–2020	$-10.7{\;}^{*}\;\left(-13.2,-8.1\right)$	$-10.7{\;}^{*}\;\left(-13.2,-8.1\right)$
40–44	2006–2020	$-6.0{\;}^{*}\;\left(-9.1,-2.9\right)$	$-6.0{\;}^{*}\;\left(-9.1,-2.9\right)$	2006–2020	$-11.4{\;}^{*}\;\left(-14.9,-7.8\right)$	$-11.4{\;}^{*}\;\left(-14.9,-7.8\right)$
45–49	2006–2020	$-5.0{\;}^{*}\;\left(-7.1,-2.9\right)$	$-5.0{\;}^{*}\;\left(-7.1,-2.9\right)$	2006–2015	−3.9(−7.8,0.2)	$-8.1{\;}^{*}\;\left(-12.1,-4.0\right)$
				2015–2020	$-15.2{\;}^{*}\;\left(-24.6,-4.6\right)$	
50–54	2006–2011	$-12.5{\;}^{*}\;\left(-20.4,-3.7\right)$	$-7.1{\;}^{*}\;\left(-12.4,-1.4\right)$	2006–2008	$-32.5{\;}^{*}\;\left(-49.3,-10.1\right)$	$-7.8{\;}^{*}\;\left(-12.1,-3.3\right)$
	2011–2017	9.0(−1.0,20.0)		2008–2017	3.2(−0.5,7.0)	
	2017–2020	$-25.5{\;}^{*}\;\left(-40.1,-7.2\right)$		2017–2020	$-19.2{\;}^{*}\;\left(-30.9,-5.5\right)$	
55–59	2006–2020	$-7.8{\;}^{*}\;\left(-9.8,-5.7\right)$	$-7.8{\;}^{*}\;\left(-9.8,-5.7\right)$	2006–2020	$-9.0{\;}^{*}\;\left(-10.4,-7.6\right)$	$-9.0{\;}^{*}\;\left(-10.4,-7.6\right)$
60–64	2006–2020	$-2.7{\;}^{*}\;\left(-5.2,-0.2\right)$	$-2.7{\;}^{*}\;\left(-52,-0.2\right)$	2006–2018	$-3.8{\;}^{*}\;\left(-5.4,-2.1\right)$	$-6.5{\;}^{*}\;\left(-10.4,-2.5\right)$
				2018–2020	−21.6(−43.1,8.2)	
65–69	2006–2015	2.5(−1.4,6.5)	−1.3(−4.5,2.1)	2006–2011	−7.1(−15.2,1.8)	−5.1(−10.5,0.6)
	2015–2020	$-7.6{\;}^{*}\;\left(-14.6,-0.0\right)$		2011–2015	6.7(−12.0,29.4)	
				2015–2020	$-11.7{\;}^{*}\;\left(-18.7,-4.1\right)$	
70–74	2006–2020	0.2(−1.4,1.8)	0.2(−1.4,1.8)	2006–2020	$-2.7{\;}^{*}\;\left(-4.1,-1.2\right)$	$-2.7{\;}^{*}\;\left(-4.1,-1.2\right)$
75–79	2006–2020	2.0(−0.4,4.5)	2.0(−0.4,4.5)	2006–2020	$-1.6{\;}^{*}\;\left(-3.1,-0.1\right)$	$-1.6{\;}^{*}\;\left(-3.1,-0.1\right)$

* Indicates statistically significant (*p* < 0.05). APC (annual percentage change); AAPC (average annual percentage change).

**Table 4:** Temporal trend analysis of age–specific mortality rates for RHD in rural Chinese residents aged 30–79 years, 2006–2020.

Age	Period	Rural Male		Period	Rural Female	
		APC (%, 95%CI)	AAPC (%, 95%CI)		APC (%, 95%CI)	AAPC (%, 95%CI)
30–34	2006–2020	$-7.3{\;}^{*}\;\left(-11.9,-2.6\right)$	$-7.3{\;}^{*}\;\left(-11.9,-2.6\right)$	2006–2020	−8.9(−12.5,−5.2)	$-8.9{\;}^{*}\;\left(-12.5,-5.2\right)$
35–39	2006–2020	$-7.1{\;}^{*}\;\left(-9.9,-4.1\right)$	$-7.1{\;}^{*}\;\left(-9.9,-4.1\right)$	2006–2008	8.0(−28.7,63.7)	$-12.3{\;}^{*}\;\left(-17.8,-6.4\right)$
				2008–2020	$-15.3{\;}^{*}\;\left(-19.6,-10.8\right)$	
40–44	2006–2020	$-8.4{\;}^{*}\;\left(-11.5,-5.1\right)$	$-8.4{\;}^{*}\;\left(-11.5,-5.1\right)$	2006–2009	−0.2(−17.3,20.4)	$-12.5{\;}^{*}\;\left(-16.7,-8.0\right)$
				2009–2020	$-15.5{\;}^{*}\;\left(-19.7,-11.1\right)$	
45–49	2006–2008	−28.1(−49.2,1.7)	−4.2(−9.9,1.9)	2006–2012	$12.1{\;}^{*}\;\left(3.2,21.7\right)$	−4.4(−14.8,7.3)
	2008–2012	17.9(−1.4,40.9)		2012–2015	−31.2(−61.0,21.4)	
	2012–2020	$-7.2{\;}^{*}\;\left(-10.6,-3.7\right)$		2015–2020	−3.9(−18.1,12.9)	
50–54	2006–2018	−1.2(−4.0,1.8)	−5.6(−13.3,2.7)	2006–2020	$-6.2{\;}^{*}\;\left(-9.0,-3.4\right)$	$-6.2{\;}^{*}\;\left(-9.0,-3.4\right)$
	2018–2020	−28.3(−62.6,37.2)				
55–59	2006–2020	$-7.9{\;}^{*}\;\left(-10.0,-5.9\right)$	$-7.9{\;}^{*}\;\left(-10.0,-5.9\right)$	2006–2009	12.5(−14.8,48.7)	$-6.9{\;}^{*}\;\left(-12.5,-0.9\right)$
				2009–2020	$-11.5{\;}^{*}\;\left(-15.7,-7.1\right)$	
60–64	2006–2011	5.7(−7.5,20.8)	−2.8(−7.7,2.3)	2006–2020	$-5.1{\;}^{*}\;\left(-7.6,-2.6\right)$	$-5.1{\;}^{*}\;\left(-7.6,-2.6\right)$
	2011–2020	$-7.3{\;}^{*}\;\left(-12.1,-2.2\right)$				
65–69	2006–2012	6.4(−5.8,20.2)	−1.5(−7.1,4.5)	2006–2012	4.2(−4.4,13.6)	−2.9(−6.8,1.2)
	2012–2020	−7.0(−13.6,0.0)		2012–2020	$-7.9{\;}^{*}\;\left(-12.5,-3.0\right)$	
70–74	2006–2010	5.2(−3.7,14.9)	−2.1(−4.6,0.5)	2006–2020	$-2.7{\;}^{*}\;\left(-4.2,-1.2\right)$	$-2.7{\;}^{*}\;\left(-4.2,-1.2\right)$
	2010–2020	$-4.8{\;}^{*}\;\left(-7.0,-2.7\right)$				
75–79	2006–2020	$-2.0{\;}^{*}\;\left(-3.9,-0.2\right)$	$-2.0{\;}^{*}\;\left(-3.9,-0.2\right)$	2006–2020	−1.6(−3.4,0.2)	−1.6(−3.4,0.2)

* Indicates statistically significant (*p* < 0.05). APC (annual percentage change); AAPC (average annual percentage change).

## Data Availability

Data for this study were obtained from the China Health Statistics Yearbook, which was compiled from the cause of death surveillance system prepared by the National Health and Family Planning Commission (closed in 2018) and by the National Health Commission of the People’s Republic of China. In addition, this yearbook collected population health data (statistics on the health level of the population and national census for historically important years).

## Abbreviations

The following abbreviations are used in this manuscript:

RHD	Rheumatic Heart Disease
APC	Annual Percentage Change
AAPC	Average Annual Percentage Change
IE	Intrinsic Estimator
APC model	Age–Period–Cohort model
CHIS	Center of Health Information and Statistics
95% CIs	95% Confidence Intervals
